# Thermal Infrared Imagery Integrated with Multi-Field Information for Characterization of Pile-Reinforced Landslide Deformation

**DOI:** 10.3390/s20041170

**Published:** 2020-02-20

**Authors:** Chang Zhou, Chunye Ying, Xinli Hu, Chu Xu, Qiang Wang

**Affiliations:** Faculty of Engineering, China University of Geosciences, Wuhan 430074, China; zhouchang@cug.edu.cn (C.Z.); cy.ying@cug.edu.cn (C.Y.); wangqiang@cug.edu.cn (Q.W.)

**Keywords:** landslide stabilizing piles, model test, thermal infrared imagery, deformation process, multi-filed information

## Abstract

Physical model testing can replicate the deformation process of landslide stabilizing piles and analyze the pile-landslide interaction with multiple field information, thoroughly demonstrating its deformation and failure mechanism. In this paper, an integrated monitoring system was introduced. The instrumentation used included soil pressure cells, thermal infrared (TIR) imagery, 3D laser scanner, and digital photography. In order to precisely perform field information analysis, an index was proposed to analyze thermal infrared temperature captured by infrared thermography; the qualitative relationship among stress state and deformation as well as thermal infrared temperature is analyzed. The results indicate that the integrated monitoring system is expected to be useful for characterizing the deformation process of a pile-reinforced landslide. Difference value of TIR temperature ($TIR_{m}$) is a useful indicator for landslide detection, and its anomalies can be selected as a precursor to landslide deformation.

## 1. Introduction

Landslides have been widely reported all over the world, especially in the Three Gorges Reservoir area in China [1,2,3,4,5]. Over 725 landslides have been stabilized with piles and other structures. The pile-landslide interaction highlights landslide evolution, which is controlled by internal and external factors and involves multi-field interaction parameters, such as stress, displacement, and temperature [6,7]. The deformation characteristics are different at different evolution stages [8,9,10]. Therefore, it is essential to identify the evolutionary stage to understand the deformation and failure process of a pile-reinforced landslide.

The physical model test is a practical and effective method [11,12,13] to reproduce the process of landslide occurrence, characterize field information, and evaluate stability in an inherently natural way [14,15,16]. Physical model tests for pile-reinforced landslide have been developed and discussed in the literature [17,18,19]. For example, Li et al. [15] suggested that the percentage of hard bedrock has a strong influence on the deformation and movement of the stabilizing pile embedded in bedrock with a hard upper and weak lower layer. However, most studies have focused on the deformation and stress of landslides and piles, analyzing its deformation and failure mechanism [20,21,22,23,24]; in addition, the analyses of temperature evolution of landslide and piles were not deep and mature enough.

In fact, objects under load such as rock and soil, with temperatures higher than absolute zero, emit infrared radiation (IRR) [25,26,27,28,29,30,31]. Luong [32] employed infrared thermography (IRT) and studied the phenomenon of IRR in the process of rock and concrete rupture. Geng et al. [33] found the existence of IRR abnormalities before rock fracturing in experiments and proposed the introduction of remote sensing into rock mechanics to form a new research field, Remote Sensing Rock Mechanics (RSRM). Over the last 20 years, this technology has made significant progress, especially in rock mechanics and unstable landslide identification [34,35,36,37,38,39]; some relatively mature approaches developed from RSRM have greatly promoted the quantitative analysis of thermal infrared (TIR) temperature [40,41,42,43,44].

Considering the feasibility of TIR temperature analysis in RSRM, IRT was recently used in landslide model tests, and some useful researches were carried out. Jin et al. [45] believed that IRT provides a new means for analyzing the deformation and failure mechanism of landslides with piles in terms of energy; Xia et al. [46] thought that IRR abnormalities could provide a reference for landslide prediction based on the research of landslide failure process using IRT. However, it is clear that IRR abnormalities and stress concentration are closely associated with geological materials under load, and perhaps that is why TIR temperature researches are mainly based on materials with a stable structure, such as rock and coal. Additionally, due to noise disturbances that inevitably exist in experimental processes, the enormous impact of environmental radiation has not been fully considered in these studies [47,48], and effective methods/indexes for TIR temperature analysis in a landslide model test have yet to be established.

In this paper, a physical model test for pile-landslide and an integrated monitoring system is constructed. According to the theoretical bases of IRR detection and particularity of soil mass, an index of TIR temperature analysis is put forward. The deformation characteristics of reinforced landslide are quantified by TIR temperature, soil pressure, and displacement. This paper provides a new method for the study of the deformation and failure mechanism of a landslide with piles.

## 2. Materials and Methods

### 2.1. Theoretical Bases of TIR Temperature Analysis

Thermal radiation, including visible light emission and IRR, is the emission of electromagnetic waves from all matter with a temperature greater than absolute zero [49]. Generally, infrared waves could be easily absorbed and scattered by the atmosphere with much attenuation, while IRT commonly operates in the infrared atmospheric window wavelengths of 3–5 μm and 8–14 μm, which offers more accuracy and reliability of temperature determination. In fact, based on thermal radiation theory, IRT is a well-established technique, and has been extensively used in temperature measurement and defect detection of various materials. 

Kirchhoff law, one of the most popular heat radiation theories, believes that the ability of an object to absorb radiation is as strong as its ability to emit it. Geotechnical materials have high absorptivity and emissivity as non-metallic materials. In this paper, the TIR temperature visualized temperature changes over the model surface. When the driving force was applied, IRT was employed to observe and capture the IRR variation. In the IRR detection process, thermograms can be obtained, and the relationship between surface temperature and radiation established by calibration so that the TIR temperature reflects the radiant energy emitted from the object. Stefan-Boltzmann law demonstrates this relationship as follows [50]:

(1) $$M=\varepsilon \sigma T^{4}$$

where M is the radiant exitance, ${\mathrm{Wm}}^{-2}\;$; ε is the emissivity of the object, 0<ε<1; σ is the Stefan–Boltzmann constant, ${\mathrm{Jm}}^{-2}K^{-4}\;$; and T is the absolute temperature of the object, K.

The Stefan-Boltzmann law states that the radiation emitted from an object is proportional to its absolute temperature to the fourth power. Precise IRR intensity for different areas is measured in this paper. Soil is non-metallic, and its radiation (over 75%) is concentrated in an infrared atmospheric window wavelength of 8–14 μm. Thus, TIR temperature is an important indicator that can reflect on the heat state and IRR variation on the surface.

According to the theoretical bases of RSRM and soil mechanics, IRT temperature variation can be induced by the thermal exchange between soil and environment, and frictional and thermoelastic effects caused by loading, respectively [27,37,50]. Here, it was assumed that IRR is divided into environment radiation (ER) and spontaneous radiation (SR). 

### 2.2. The Effective Index of TIR of Landslide Stabilizing Piles

Ma et al. [51] introduced the average value of temperature changes ($\bar{\Delta TIR}$) to characteristic the failure of a landslide model. However, in a landslide stabilizing pile model, temperature variability is large because of the pile and soil; the monitoring results also indicate that the $\bar{TIR}$ could not descript the deformation process of a pile-reinforced landslide. Therefore, the maximum and minimum values of temperature changes ($TIR_{max}$, $TIR_{min}$) for the region of interest are introduced. The $TIR_{max}$, $TIR_{min}$, and $\bar{TIR}$ are named as 3M index. Thus, denote a pixel matrix {T(x, y)} at certain timing, where:

(2) $${T_{x,y}|}_{t}={T_{x,y}^{ER}|}_{t}+{T_{x,y}^{SR}|}_{t}$$

$\Delta TIR_{max}$ for each pixel matrix {T(x, y)} at certain timing represents the temperature characteristics of the vast majority of pixels in each region, and is calculated by

(3) $${TIR_{max}|}_{t}={\frac{1}{n}\sum T_{x,y}|}_{t}={\frac{1}{n}\sum T_{x,y}^{ER}|}_{t}+{\frac{1}{n}\sum T_{x,y}^{SR}|}_{t}$$

While stress in the model increases, *SR* caused by the frictional effects is enhanced, so that $TIR_{max}\;$ at a pixel $\left(x_{1},y_{1}\right)$ occurs and can be obtained by:

(4) $${TIR_{max}|}_{t}=Max\left\{T_{x,y}\right\}={T_{x_{1},y_{1}}^{ER}+T_{x_{1},\;\;y_{1}}^{SR}|}_{t}$$

While incompatible deformation or cracks develop on the model surface, the thermal exchange between soil and environment is hindered and ER weakens, which results in $TIR_{min}$ at certain other pixels $\left(x_{2},y_{2}\right)$ that can be obtained by:

(5) $${TIR_{min}|}_{t}=Min\left\{T_{x,y}\right\}={{\left(T-\Delta T\right)}_{x_{2},y_{2}}^{ER}|}_{t}$$

Actually, there is no positive connection between the two, which describes the distinct information about model evolution. In addition, $T^{ER}$ is contained in all of the 3M indexes, and its impact on the TIR temperature should be eliminated. Suppose $\left\{T_{x,y}^{ER}\right\}$ is equal in each region, and denotes the maximum range of {T(x, y)} as difference value of TIR temperature ($TIR_{m}$), where:

(6) $${TIR_{m}|}_{t}={TIR_{max}-TIR_{min}|}_{t}={T_{x_{1},y_{1}}^{ER}+T_{x_{1},\;\;y_{1}}^{SR}-{\left(T-\Delta T\right)}_{x_{2},y_{2}}^{ER}|}_{t}={T_{x_{1},\;\;y_{1}}^{SR}+\Delta T_{x_{2},y_{2}}^{ER}|}_{t}$$

In the regions without crack, $TIR_{m}$ degenerates to:

(7) $${TIR_{m}|}_{t}={T_{x_{1},\;\;y_{1}}^{SR}|}_{t}$$

$TIR_{m}$ indicator could manifest IRR abnormalities resulting from *SR*, and also indicate the degree of deformation of slope surface. Thus, within a certain time and space, $TIR_{max}$ and $TIR_{min}$ can feature high or low *TIR* temperature abnormities caused by the stress field and displacement field. Namely, as satisfied information integration that is almost free from *ER*, this index has a relatively definite meaning.

According to the thermo-mechanical coupling theory [52], infrared radiation temperature T consists of three parts:

(8) $$T=T_{1}+T_{2}+T_{3}$$

where $T_{1}$ is infrared radiation temperature caused by the thermal-elastic effect; $T_{2}$ is caused by newly produced cracks of a model; $T_{3}$ is caused by friction heat.

### 2.3. Landslide Stabilizing Piles Model

**Figure 1:**
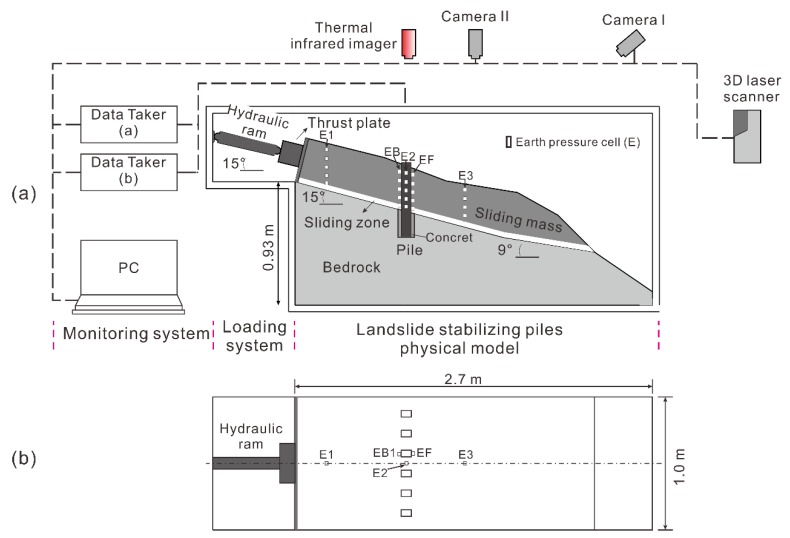
Landslide stabilizing piles model testing system from (**a**) front view and (**b**) side view.

The landslide stabilizing piles model test was described by Hu et al. (2019) [53]. The model was constructed on a rigid steel frame, which is 2.7 m long, 1.0 m wide, and 1.5 m high (Figure 1). The model includes three parts: loading system, monitoring system, and landslide stabilizing piles model (Figure 1a). The landslide stabilizing piles model consists of a sliding mass, sliding zone, and six flexible piles. The geometry of the landslide model was designed by taking a typical colluvial landslide located in the Three Georges as the prototype. The sliding zone dips 9° and then changes to 15° at an elevation of 51 cm. The average thickness of the sliding mass and sliding zone is 0.35 m and 4cm, respectively. The material of the model was developed by hundreds of proportioning tests [54]. The geometry parameters of the model and the properties of the material are shown in Table 1.

A consistent load force and displacement are applied to the model to simulate the evolution progress of pile-reinforced landslide. The load is parallel to the sliding zone inclination. Stepwise loading is designed to ensure that the load was wholly transmitted into the model. It is also useful for analyzing the deformation of the model under each step. In this paper, during each step, the load was steadily increased by 500 N over 30 min and then maintained for another 30 min.

### 2.4. Monitoring System

#### 2.4.1. Thermal Infrared Temperature Monitoring

H-2630 IRT is a device that converts energy into electrical signals (Table 2). It has an accuracy of ±2% of reading, temperature resolution of 0.04 °C, and infrared image resolution of 640 × 480 pixels, and adopts environment temperature compensation. The emissivity value and reflectivity of sliding mass soil are 0.90–0.98, 0.1–0.2, respectively [55]. The emissivity of the pile is 0.855 [56]. The scene was captured from a distance of approximately 1 m. In order to reduce the effect of changing cloud shadowing and wind, the testing was done indoors, and ambient temperature and relative humidity were recorded. Besides, to compensate for inaccuracy due to the distance of the observed objects, ambient temperature, and relative humidity, we developed an index named difference value of IRT temperature (∆$TIR_{m}$) to accurately describe the change of the thermal infrared temperature in the model. The parameters of the IRT are shown in Table 2. The IRT was equipped at the upper side of the model to monitor the temperature of the model surface around piles (Figure 1).

#### 2.4.2. Surface Deformation

The deformation of the model surface was recorded using two video cameras and a 3D laser scanner. The white spherical pushpin (10 mm diam) served as monitoring points, and they were installed into the model surface and pile heads at equal intervals of horizontal distance. The particle image velocimetry (PIV) (Baba and Peth, 2012) was used to measure the location of the monitoring points. The changes in monitoring locations were analyzed to obtain movement of the model surface. The RIEGL VZ-400 3D laser scanner used in this paper has a precision of 2 mm and was placed at approximately 1 m in front of the testing frame (Figure 1). The scanner operated every five minutes.

#### 2.4.3. Earth Pressure

In this paper, 34 earth pressure cells were used. The cells had a vertical spacing of 50 mm in the same section. Nineteen earth pressure cells were instrumented along the center axis of the landslide model (Figure 1a). In the reinforced landslide, 15 of these were symmetrically set in the uphill and downhill sides of pile 4 and pile 3 (Figure 1b). The results of the monitoring help understand the pile-landslide interaction.

## 3. Results

### 3.1. Deformation Characteristics of Landslide Stabilizing Piles

**Figure 2:**
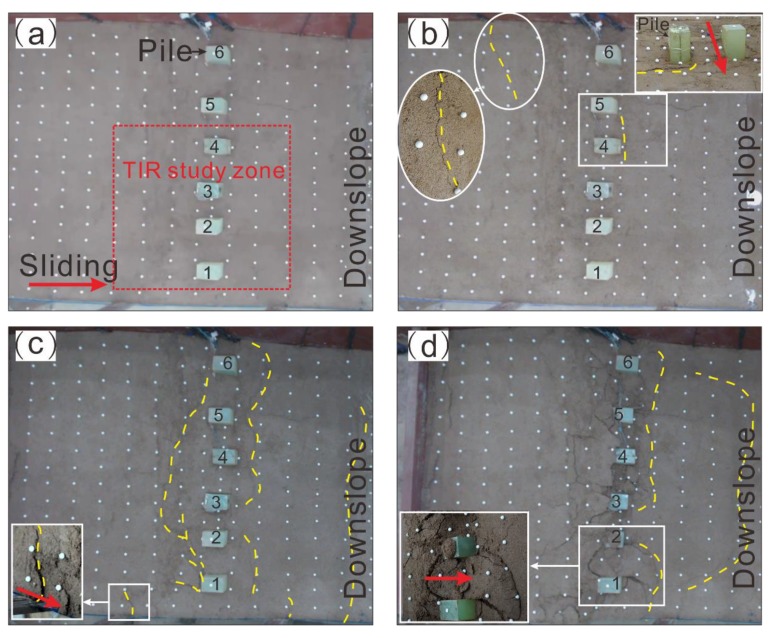
Deformation characteristics of the model surface at (**a**) 0.0 h, (**b**) 6.5 h, (**c**) 10.5 h, (**d**) 12 h.

During the tests, the surface deformation of the model was recorded using video cameras. Photographs were also taken to visualize the deformation characteristics of the model at different times (Figure 2).

During the initial stage of loading from 0 to 6 h, the landslide surface had no obvious deformation, except for the soil near the thrust plate, which was progressively mobilized. As the loading increased from 6 to 12 h, surface deformation occurred throughout the model. Several cracks were generated at the uphill side of the piles (Figure 2b). During the eleventh loading cycle (t = 710 min), the sliding mass at the uphill side of the piles was uplifted about 2 cm, and piles had notable deformation (Figure 2c). As the load further increased, the cracks at the downhill side of the piles gradually expanded in length, and the sliding mass was uplifted (Figure 2d). 

**Figure 3:**
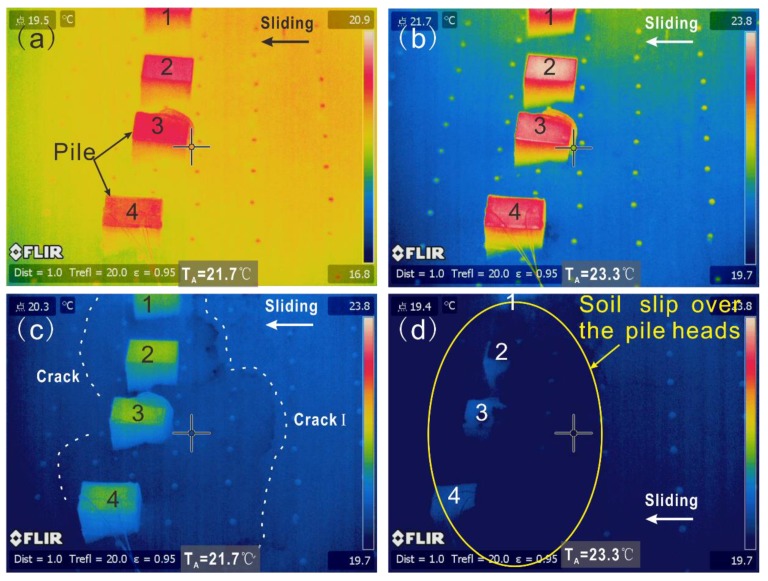
TIR of the model and landslide (**a**) 0.0 h, (**b**) 6.5 h, (**c**) 10.5 h, (**d**) 12 h. $T_{A}$ is ambient temperature.

The TIR monitoring results are shown in Figure 3. It was found that the TIR was obviously affected by environment radiation (ER). The TIR in the pile heads was more extensive than that in the soil. Before the test, the TIR in the model surface had no obvious change (Figure 3a). As the load increased, energy accumulate caused increase of the TIR in the upslope. When t = 6.5 h, ambient temperature ($T_{A}$) was 23.3 °C, and the average value of the soil was about 21.7 °C (Figure 3b). The difference value of the pile-soil was 1.6 °C which is larger than that before testing. It was inferred that the TIR gradually changed. As the loading increased, the anomaly of the TIR showed zonal distribution around the piles (Figure 3c) at the location of the cracks (Figure 2c). Moreover, when part of the sliding mass slipped over the pile heads (Figure 2d), the TIR behind the piles was smaller than that of the other parts (Figure 3d).

### 3.2. Characteristics of TIR around Piles

**Figure 4:**
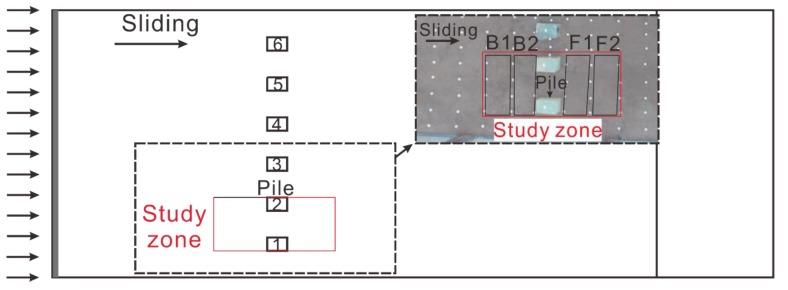
Study zone of the model. The zone is divided into five areas: B1, B2 are located upstream of the pile, F1 and F2 are located downstream of the pile. Pile 2 is selected to study the temperature characteristics of piles.

In order to analyze the deformation characteristics of a landslide with piles during the evolution process, the model surface around pile 1 and pile 2 was selected as the study zone, which was divided into five parts: piles, B1 and B2 located behind the piles, F1 and F2 situated in front of the piles (Figure 4). The maximum, minimum, and average values (3M indicators) of the TIR in those five parts were counted. 

**Figure 5:**
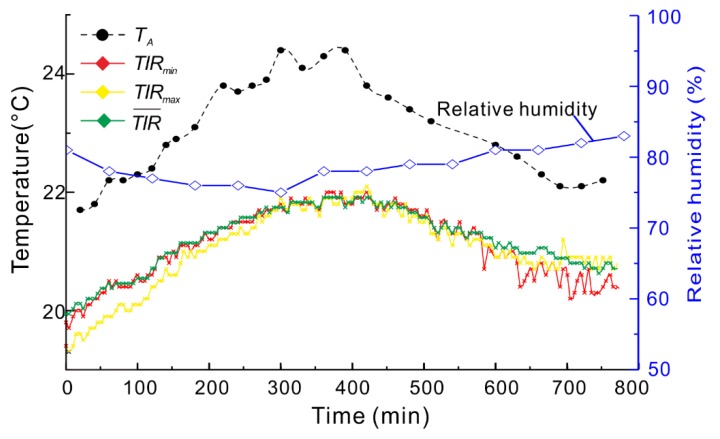
3M index of the study zone B2 and ambient temperature ($T_{A}$) and relative humidity versus time. $TIR_{min}$, $TIR_{max}$, $\bar{TIR}$ is the minimum, maximum, and average value of temperature for the study region.

3M indicators in the B2 and ambient temperature are shown in Figure 5. During 0–300 min, the ambient temperature gradually increased, and then decreased from 400–800 min. The same change in 3M indicators was observed during the testing. TIR gradually increased during 0–400 min and then decreased, which is similar to the ambient temperature change. Therefore, 3M indicators are correlated with ambient temperature; thus, those indicators could not be used to analyze the deformation characteristics of the landslide.

**Figure 6:**
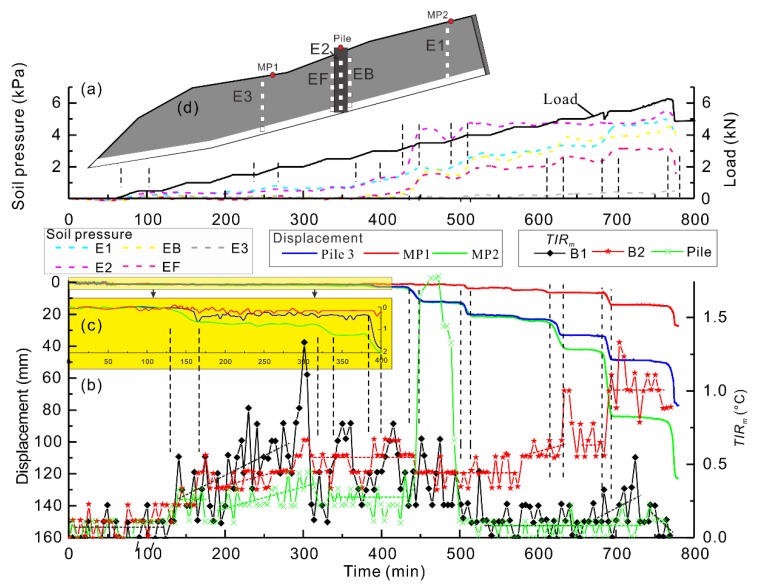
Time-series of load (black line), soil pressures (dashed line) (**a**), displacement (solid line), and $TIR_{m}$ (solid line with symbol) (**b**,**c**) is the displacement of the model during 0–400 min, showing detailed deformation characteristics. The vertical black dashed lines identify rapid change in displacement and soil pressure curves.

Based on the monitoring results, multi-field data are compared (Figure 6). During 0–360 min, the load applied in the model was less than 3274 N, and only small displacements (<3 mm) were induced in the model (Figure 6c). The displacements accelerated in a step-like manner, consistent with the loading stages on the thrust plate during 360–780 min. The displacement of the pile head was similar to the MP2, but after 610 min, the difference of the displacement between pile and MP2 gradually increased. Moreover, MP1, located in the downstream side of the piles also had a noticeable change. Therefore, it was inferred that the piles were progressively separated from the upslope, and the stress was transferred into the downslope. Soil pressure also had a similar change. The average value of soil pressure in each section was calculated (Figure 6a). It also raised an inconspicuous step-like manner consistent with the loading stages on the thrust plate. The soil pressure cell closest to the thrust plate at E1 was the first to respond to the thrust loading. The soil pressure cell at the uphill (EB) and downhill (EF) side of the piles responded at 370 min. The soil pressure around the piles (E2, EB, EF) rapidly increased after 400 min, when the piles and the model had obvious deformation. Besides, soil pressure at E2 is larger than that at E1, which could be caused by soil arching. Only small soil pressure (<0.5 kPa) was induced in the lower part of the landslide model (E3).

The $TIR_{m}$ in the reign of interest began to increase at 130 min. During 285–301 min, the $TIR_{m}$ in B1 rapidly grew from 0.2 °C to 1.4 °C, but the displacement had small changes. Thus, we thought the increase of $TIR_{m}$ was mainly caused by $T_{3}$ and $T_{1}$, because $\Delta T_{2}$ was negative, and the soil pressures increased by 0.4 kPa, which means that $\Delta T_{1}$ was positive, but small. Then, $TIR_{m}$ decreased to 0.1 °C within 12 min, and the displacement of MP2 had an apparent increase; thus, we thought some fractures were generated before the landslide movement, which caused the decrease of $TIR_{m}$. During 313–475 min, it had large fluctuation and then kept stable at around 0.1 °C during 530 min–560 min. When the difference of the displacement between pile and MP2 rapidly increased, the $TIR_{m}$ in B1 increased again after 560 min and reached a maximum value at 725 min. The $TIR_{m}$ in B2 had a similar change with B1 during 0–500 min. Then, the average value stayed stable. At 635 min and 685 min, when the displacement rapidly increased, the $TIR_{m}$ in B2 also rapidly increased, because the movement of the model induced friction heat ($\Delta T_{3}$) and the soil pressures increased ($\Delta T_{1}$). $TIR_{m}$ in the pile head also had similar changes as that in B1, but smaller. After 440 min, when the piles had obvious displacement and soil pressure around the piles rapidly increased, the $TIR_{m}$ in the pile head rapidly increased by 1.2 °C within 6 min. It was inferred that the force acting on piles rapidly increased; thus, the increase of $TIR_{m}$ could have been caused by $\Delta T_{1}$. Therefore, anomalies of $TIR_{m}$ in the model surface occurred when the soil pressure rapidly increased, caused by the acceleration of displacement. In conclusion, stress, displacement, and TIR had good correlation, validating the effectiveness of the $TIR_{m}$ indicator.

## 4. Discussion

In order to verify the non-randomness of $TIR_{m}$, the rescaled range method was used to analyze the dual nature of regularity and randomness, and calculate the long-term correlation of this time series [57]; thus, its Hurst exponent (H) was obtained by the method proposed by Xu et al. [58]. The value of Hurst exponent (H) varies between 0 and 1. When 0 < H < 0.5, it means a completely uncorrelated series. When H > 0.5, it means that the regularity of the series is stronger. The calculated results of the Hurst exponent of B1, B2, and Pile is 0.847, 0.813, 0.750, respectively. This demonstrates that its long-term positive correlation is quantitatively pretty dramatic, or, relative to its randomness, $TIR_{m}$ has more regularity.

Landslides caused the friction and collisions of soil particles, which induce energy change. The greater the deformation rate, the greater the change in stress, and the energy in landslide changes. However, differing from landslide deformation, the change of energy includes two stages of energy accumulation and release, and it is a slow process. Therefore, the $TIR_{m}$ required a longer time to rapidly increase versus the model displacement. In summary, during the first stage, the TIR gradually increased caused by the increase of stress ($T_{1})$ and friction heart ($T_{3}$), and the $TIR_{m}$ rapidly decreased in the model caused by $T_{2}$, the generation of cracks [51]. In the second stage, the $TIR_{m}$ change was caused by $T_{1}$, $T_{2}$ and $T_{3}$. The sudden large displacement caused the change of friction heart and generation of cracks. Moreover, the increase of soil pressure caused the $T_{1}$ to increase. Therefore, when a sudden large displacement occurred, $TIR_{m}$ had a large change, which could be selected as a precursor of landslide with piles deformation.

Compared with B1, $TIR_{m}$ in B2 gradually increased after the model had visible deformation, and accelerated in a step-like manner consistent with the rapid displacement increase. It could be because the soil arching effect exists around piles, causing stress concentration around the piles. As a result, soil pressure behind the piles is more significant than that in the upper part of the model. Due to soil arching, the stress mainly concentrates on the soil behind the piles [59], causing temperature difference ($TIR_{m}$) for B2 to be larger than that for B1, where stress is more uniform. Therefore, the $TIR_{m}$ for B2, closer to the piles, had an obvious response compared to that for B1. Therefore, the $TIR_{m}$ could be used to analyze the pile-soil interaction.

Landslide evolution is a complex multi-fields dynamic process that involves the interaction of seepage, stress, deformation, and temperature fields [6]. The results of model tests show that TIR had good coordination with stress and displacement. A higher resolution of IRT and an advanced index for the TIR temperature analysis may be included in further study.

## 5. Conclusions

To understand the deformation process of landslides with piles and quantify the thermal characteristics, an integrated monitoring system was constructed, including TIR imagery, 3D laser scanner, high-speed cameras, soil pressure cell, and other instrumentation to obtain multi-field information for the pile-reinforced landslide deformation process. The value of difference between maximum and minimum TIR temperature ($TIR_{m}$) for the region of interest was utilized to appreciate the temperature characteristics and identify the anomalies associated with the deformation and stress of the landslide. The results show that $TIR_{m}$ was able to decrease the effect of atmospheric attenuation and is promising for deformation characterization of the landslide with piles. TIR temperature anomalies, such as cracks and heaved area of the sliding mass, occurred in the landslide deformation area. The $TIR_{m}$ in the upstream side of the piles had an obvious response to anomalies in the displacements and soil pressures, especially near the piles. During the landslide deformation, energy was gradually accumulated in the piles, and when the piles had obvious deformation, the $TIR_{m}$ rapidly decreased, caused by energy dissipation. $TIR_{m}$ can be a useful indicator of the temperature field of landslide stabilizing piles. The rapid increase in $TIR_{m}$ can be selected as a precursor for landslides with pile deformation.

## Figures and Tables

**Table 1:** Main features of the landslide stabilizing piles model.

Object	Parameters of Materials	Geometric Parameters
Sliding zone	Cohesion(kPa): 5.5 to 6.0 Friction angle (°): 17.9 to 18.2 Density (kN/m^3^): 17.1	Thickness: 4 cm
Sliding mass	Cohesion(kPa): 3.5 to 4.2 Friction angle (°): 23.6 to 24.1 Density (kN/m^3^): 22.1	Thickness: 35 ± 1 cm Width: 100 cm Length of the upstream of piles: 80 cm Length on the downstream of piles: 139 cm Length of sliding mass: 226 cm
Pile	Elastic modulus (GPa): 0.03	Pile cross section: 5 × 7.5 cm Cantilever length: 37 ± 1 cm Embedded Length: 19 ± 1 cm Horizontal spacing: 15 cm Amount: 6

**Table 2:** Main features of the instrumentation monitoring units.

Instrumentation	Specifications	Photograph
NEC-H2630	Measuring range (°C): −40 to 500 Resolution (°C): 0.04 °C or better (at 30 °C, ∑16 Accuracy: ±2% of reading Spectral range (μm): 8 to 13 Focusing range: 30 cm to infinity Thermal image pixels: 640 (H) × 480 (V)	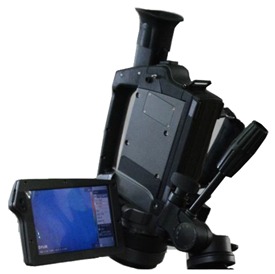
RIEGL VZ-400	Maximum pulse repetition rate (PRR) (kHz): 300 (high-speed model) Effective measurement rate (meas./s): 122,000 (high-speed model) Minimum range (m): 1.5 Maximum range (m): 600 (long-range model)/350 (high-speed model) Accuracy|Precision (mm): 5/3 Laser wavelength (nm): 1550 Laser beam divergence (mrad): 0.3	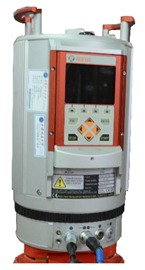
Giga View	Resolution: 1280 × 1024 Frame-rates (fps): 50 to 17,000 Shutter: 1/50–1/100,000 Sensor: 10-bit mono or 24-bit color	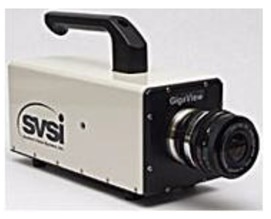
TXR soil pressure cell	Diameter: 2 cm ± 1 mm Measuring range (MPa): 0 to 0.1 Resolution (%F·S): ≤0.08 Temperature measuring range (°C): −25 to 60	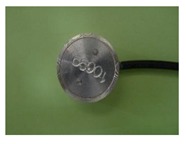

## References

[1] Wu S., Shi L., Wang R., Tan C., Hu D., Mei Y., Xu R. (2001). Zonation of the landslide hazards in the forereservoir region of the Three Gorges Project on the Yangtze River. Eng. Geol..

[2] Wang F., Zhang Y., Huo Z., Matsumoto T., Huang B. (2004). The July 14, 2003 Qianjiangping landslide, Three Gorges Reservoir, China. Landslides.

[3] Huang B., Yin Y., Liu G., Wang S., Chen X., Huo Z. (2012). Analysis of waves generated by Gongjiafang landslide in Wu Gorge, Three Gorges Reservoir, on November 23, 2008. Landslides.

[4] Xu G., Li W., Yu Z., Ma X., Yu Z. (2015). The 2 September 2014 Shanshucao landslide, Three Gorges Reservoir, China. Landslides.

[5] Sun G., Zheng H., Tang H., Dai F. (2016). Huangtupo landslide stability under water level fluctuations of the Three Gorges Reservoir. Landslides.

[6] Hu X., Tan F., Tang H., Zhang Y., Zhang G., Su A., Xu C., Xiong C. (2017). In-situ monitoring platform and preliminary analysis of monitoring data of Majiagou landslide with stabilizing piles. Eng. Geol..

[7] Zhang Y., Hu X., Tannant D.D., Zhang G., Tan F. (2018). Field monitoring and deformation characteristics of a landslide with piles in the Three Gorges Reservoir area. Landslides.

[8] Tang H., Li C., Hu X., Su A., Wang L., Wu Y., Criss R., Xiong C., Li Y. (2015). Evolution characteristics of the Huangtupo landslide based on in situ tunneling and monitoring. Landslides.

[9] Crosta G.B., Agliardi F., Rivolta C., Alberti S., Dei Cas L. (2017). Long-term evolution and early warning strategies for complex rockslides by real-time monitoring. Landslides.

[10] Gu D.M., Huang D., Yang W.D., Zhu J.L., Fu G.Y. (2017). Understanding the triggering mechanism and possible kinematic evolution of a reactivated landslide in the Three Gorges Reservoir. Landslides.

[11] Luo X.Q., Sun H., Tham L.G. (2010). Landslide model test system and its application on the study of Shiliushubao landslide in Three Gorges Reservoir area. Soils Found..

[12] Iverson R.M. (2015). Scaling and design of landslide and debris-flow experiments. Geomorphology.

[13] Zhang Z., Wang T., Wu S., Tang H., Liang C. (2017). Investigation of dormant landslides in earthquake conditions using a physical model. Landslides.

[14] Jia G.W., Zhan T.L.T., Chen Y.M., Fredlund D.G. (2009). Performance of a large-scale slope model subjected to rising and lowering water levels. Eng. Geol..

[15] Li C., Wu J., Tang H., Hu X., Liu X., Liu T., Wang C., Zhang Y. (2016). Model testing of the response of stabilizing piles in landslides with upper hard and lower weak bedrock. Eng. Geol..

[16] He C., Hu X., Tannant D.D., Tan F., Zhang Y., Zhang H. (2018). Response of a landslide to reservoir impoundment in model tests. Eng. Geol..

[17] Wang L., Zhang G. (2014). Centrifuge model test study on pile reinforcement behavior of cohesive soil slopes under earthquake conditions. Landslides.

[18] Zhu H., Shi B., Yan J., Zhang J., Wang J. (2015). Investigation of the evolutionary process of a reinforced model slope using a fiber-optic monitoring network. Eng. Geol..

[19] Hussien M.N., Tobita T., Iai S., Karray M. (2016). Soil-pile-structure kinematic and inertial interaction observed in geotechnical centrifuge experiments. Soil Dyn. Earthq. Eng..

[20] Pan J.L., Goh A.T.C., Wong K.S., Teh C.I. (2002). Ultimate soil pressures for piles subjected to lateral soil movements. J. Geotech. Geoenviron. Eng..

[21] Zornberg J.G., Arriaga F. (2003). Strain distribution within geosynthetic-reinforced slopes. J. Geotech. Geoenviron..

[22] Zomorodian S.M.A., Dehghan M. (2011). Lateral resistance of a pile installed near a reinforced slope. Int. J. Phys. Model. Geotech..

[23] Zhu H.H., Shi B., Yan J.F., Zhang J., Zhang C.C., Wang B.J. (2014). Fiber Bragg grating-based performance monitoring of a slope model subjected to seepage. Smart Mater. Struct..

[24] Guo W.D. (2015). Nonlinear response of laterally loaded rigid piles in sliding soil. Can. Geotech. J..

[25] Brady B.T., Rowell G.A. (1986). Laboratory investigation of the electrodynamics of rock fracture. Nature.

[26] Geng N., Yu P., Deng M., Cui C., Luo Z. (1998). The simulated experimental studies on cause of thermal infrared precursor or earthquakes. Earthquake.

[27] Wu L.X., Cui C.Y., Geng N.G., Wang J.Z. (2000). Remote sensing rock mechanics (RSRM) and associated experimental studies. Int. J. Rock Mech. Min..

[28] Tronin A.A., Hayakawa M., Molchanov O.A. (2002). Thermal IR satellite data application for earthquake research in Japan and China. J. Geodyn..

[29] Wu Y.H., Wu L.X., Wu H.P., Liu S.J. (2002). Changes in infrared radiation with rock deformation. Int. J. Rock Mech. Min..

[30] Ouzounov D., Liu D., Chunli K., Cervone G., Kafatos M., Taylor P. (2007). Outgoing long wave radiation variability from IR satellite data prior to major earthquakes. Tectonophysics.

[31] Hayakawa M., Liu J., Hattori K., Telesca L. (2009). Electromagnetic phenomena associated with earthquakes and volcanoes preface. Phys. Chem. Earth Parts A/B/C.

[32] Luong M.P. (1990). Infrared thermovision of damage processes in concrete and rock. Eng. Fract. Mech..

[33] Geng N., Cui C., Deng M. (1992). Remote sensing detection on rock fracturing experiment and the beginning of remote sensing rock mechanics. Acta Seismol. Sin..

[34] Wu L.X., Wang J.Z. (1998). Infrared radiation features of coal and rocks under loading. Int. J. Rock Mech. Min..

[35] Wu Y.H., Wu L.X., Liu S.J., Wang C.Y. (2006). Precursors for rock fracturing and failure—Part I: IRR image abnormalities. Int. J. Rock Mech. Min..

[36] Zhao Y.X., Jiang Y.D. (2010). Acoustic emission and thermal infrared precursors associated with bump-prone coal failure. Int. J. Coal Geol..

[37] He M.C., Gong W.L., Zhai H.M., Zhang H.P. (2010). Physical modeling of deep ground excavation in geologically horizontal strata based on infrared thermography. Tunn. Undergr. Space Technol. Inc. Trenchless Technol. Res..

[38] Li Z.H., Yin S., Niu Y., Cheng F.Q., Liu S.J., Kong Y.H., Sun Y.H., Wei Y. (2018). Experimental study on the infrared thermal imaging of a coal fracture under the coupled effects of stress and gas. J. Nat. Gas Sci. Eng..

[39] Luo L., Ma W., Zhao W., Zhang Y., Zhang Z., Zhang M., Ma D., Zhou Q. (2018). UAV-based spatiotemporal thermal patterns of permafrost slopes along the Qinghai–Tibet Engineering Corridor. Landslides.

[40] Wu Y.H., Wu L.X., Liu S.J., Wang C.Y. (2006). Precursors for rock fracturing and failure—Part II: IRR T-Curve abnormalities. Int. J. Rock Mech. Min..

[41] Wang C.L., Lu H., Lu Z.J., Liu L., Chuai X.S. (2016). Predicting points of the infrared precursor for limestone failure under uniaxial compression. Int. J. Rock Mech. Min..

[42] Ma L.Q., Sun H., Zhang Y., Zhou T., Li K., Guo J.S. (2016). Characteristics of infrared radiation of coal specimens under uniaxial loading. Rock Mech. Rock Eng..

[43] Ma L.Q., Sun H. (2018). Spatial-temporal infrared radiation precursors of coal failure under uniaxial compressive loading. Infrared Phys. Technol..

[44] Zhang F., Zhang X.L., Li Y.J., Tao Z.G., Liu W.F., He M.C. (2018). Quantitative description theory of water migration in rock sites based on infrared radiation temperature. Eng. Geol..

[45] Jin L., Hu X., Tan F., He C., Zhang H., Zhang Y. (2016). Model test of soil arching effect of anti-slide piles based on infrared thermal imaging technology. Rock Soil Mech..

[46] Xia H., Hu X., Tang H., Yong R., Ma J. (2017). Application of infrared thermal radiation imaging technology to landslide physical model test. Rock Soil Mech..

[47] Liu L., Chen G., Liu P., Chen S., Ma J. (2004). Infrared measurement system for rock deformation experiment. Seismol. Geol..

[48] Sun H., Ma L.Q., Adeleke N., Zhang Y. (2017). Background thermal noise correction methodology for average infrared radiation temperature of coal under uniaxial loading. Infrared Phys. Technol..

[49] Planck M. (1959). The Theory of Heat Radiation.

[50] Boltzmann L. (1884). Ableitung des stefan’schengesetzes, betreffend die abhängigkeit der wärmestrahlung von der temperaturaus der electromagnetischen lichttheorie. Ann. Physik-Berl..

[51] Thomson M. (1852). On the dynamical theory of heat. Philos. Mag..

[52] Ma J.W., Niu X.X., Liu X., Wang Y.K., Wen T., Zhang J. (2020). Thermal Infrared Imagery Integrated with Terrestrial Laser Scanning and Particle Tracking Velocimetry for Characterization of Landslide Model Failure. Sensors.

[53] Sun X.M., Chen F., Miao C.Y., Song P., Li G., Zhao C.W., Xia X. (2018). Physical modeling of deformation failure mechanism of surrounding rocks for the deep-buried tunnel in soft rock strata during the excavation. Tunn. Undergr. Space Technol..

[54] Hu X.L., Chang Z., Xu C., Liu D., Wu S., Li L. (2019). Model tests of the response of landslide-stabilizing piles to piles with different stiffness. Landslides.

[55] Xu C., Hu X.L., He C.C., Xu Y., Zhou C. (2018). Development and application of similar material for reservoir landslide model test. Rock Soil Mech..

[56] Xiao S., Zhang T., Wang Z.Y., Liu P.R. (2016). The Research on the Infrared Emissivity of Water-borne Polyurethane Coatings. Infrared Technol..

[57] Azemati A., Khorasanizadeh H., Hadavand B.S. (2017). Study on Radiation Properties of Polyurethane/Nano Zirconium Oxide Nanocomposite Coatings. Mater. Sci. Forum.

[58] Xu W.Y., Meng Q.X., Wang R.B., Zhang J.C. (2016). A study on the fractal characteristics of displacement time-series during the evolution of landslides. Geomat. Nat. Hazards Risk.

[59] Tang H., Hu X., Xu C., Li C., Yong R., Wang L. (2014). A novel approach for determining landslide pushing force based on landslide-pile interaction. Eng. Geol..

